# Data of pink-beam serial synchrotron crystallography at the Pohang Light Source II

**DOI:** 10.1016/j.dib.2023.109811

**Published:** 2023-11-17

**Authors:** Yongsam Kim, Ki Hyun Nam

**Affiliations:** a Pohang Accelerator Laboratory, Pohang University of Science and Technology, 37673, Republic of Korea; b College of General Education, Kookmin University, Seoul 02707, Republic of Korea

**Keywords:** Serial synchrotron crystallography, Pink-beam, Room-temperature, Syringe, Lysozyme

## Abstract

Serial synchrotron crystallography (SSX) helps to determine the room-temperature structure of macromolecules with minimal radiation damage. Pink-beam X-ray provides more photon flux than a monochromatic beam, which can increase the diffraction intensity of crystal samples and reduce the issue of partial reflection measurement compared with a monochromatic beam. The demonstration of pink-beam SSX at the 1C beamline at the Pohang Light Source II (PLS-II) was previously reported. The Bragg peaks observed in SSX diffraction data using a pink-beam exhibited a slightly stretched shape, unlike that from a monochromatic beam. Therefore, it is necessary to develop an indexing algorithm that can efficiently process the Bragg peak generated by pink-beam SSX. Therefore, the collected pink-beam SSX diffraction data can be tentatively used to develop an indexing program for Bragg peaks generated using the pink-beam. In this study, detailed information on the diffraction data of pink-beam SSX at PLS-II was reported to access the raw data and process the information.

Specifications Table

**Table:** 

Subject	Biological sciences
Specific subject area	Structural biology
Data format	Raw, Analyzed
Type of data	X-ray diffraction data, Table, Image, Graph, Figure
Data collection	Synchrotron: Pohang Light Source II (PLS-II) Beamline: 1C X-ray wavelength: 15.48 keV X-ray bandwidth (ΔE/E): 1.2 % Photon flux: ∼5 × 10^12^ photons/second Beam size: 70 × 80 μm (full width at half maximum, vertical x horizontal) Sample delivery: viscous medium-based injection via syringe and syringe pump Sample flow rate: 1 μL/min Detector: Pilatus3S 2 M (DECTRIS). Data acquisition: 10 or 20 Hz Data collection temperature: 26 ± 0.5°
Data source location	Institution: Kookmin University City/Town/Region: Seoul Country: Republic of Korea
Data accessibility	1. Raw data diffraction images Repository name: ZENODO 1) Data 1 (LysEdge50): X-ray exposed to the edge of the injection stream with an X-ray exposure time of 50 ms - Digital Object Identifier: https://doi.org/10.5281/zenodo.7240990 - Direct URL to data: https://zenodo.org/records/7240990 2) Data 2 (LysCenter50): X-ray exposed to the center of the injection stream with an X-ray exposure time of 50 ms. - Digital Object Identifier: https://doi.org/10.5281/zenodo.7239100 - Direct URL to data: https://zenodo.org/records/7239100 3) Data 3 (LysEdge100): X-ray exposed to the edge of the injection stream with an X-ray exposure time of 100 ms - Digital Object Identifier: https://doi.org/10.5281/zenodo.7242977 - Direct URL to data: https://zenodo.org/records/7242977 4) Data 4 (LysCenter100): X-ray exposed to the center of the injection stream with an X-ray exposure time of 100 ms. - Data identification number: https://doi.org/10.5281/zenodo.7242973 - Direct URL to data: https://zenodo.org/records/7242973 2. Structure factor and coordinate Repository name: Protein Data Bank 1) Data 1: X X-ray exposed to the edge of the injection stream with an X-ray exposure time of 50 ms - PDB code: 8H8T - Data identification number: https://doi.org/10.2210/pdb8H8T/pdb - Direct URL to data: https://www.rcsb.org/structure/8H8T 2) Data 2: X-ray exposed to the center of the injection stream with an X-ray exposure time of 50 ms. - PDB code: 8H8U - Data identification number: https://doi.org/10.2210/pdb8H8U/pdb - Direct URL to data: https://www.rcsb.org/structure/8H8U
	3) Data 3: X-ray exposed to the edge of the injection stream with an X-ray exposure time of 100 ms - PDB code: 8H8V - Data identification number: https://doi.org/10.2210/pdb8H8V/pdb - Direct URL to data: https://www.rcsb.org/structure/8H8V 4) Data 4: X-ray exposed to the center of the injection stream with an X-ray exposure time of 100 ms. - PDB code: 8H8W - Data identification number: https://doi.org/10.2210/pdb8H8W/pdb - Direct URL to data: https://www.rcsb.org/structure/8H8W
Related research article	Y. Kim, K.H. Nam, Pink-Beam Serial Synchrotron Crystallography at Pohang Light Source II, Crystals (2022) [1]https://doi.org/10.3390/cryst12111637

## 1 Value of the Data

• The first pink-beam serial synchrotron crystallography experiment at Pohang Light Source II was conducted.
• The first diffraction data of pink-beam serial synchrotron crystallography at Pohang Light Source II were collected.
• Four room-temperature structures of lysozyme using pink-beam serial synchrotron crystallography were determined.
• Data collection strategies were reported based on four different diffraction datasets.

## 2 Data Description

A broad energy bandwidth pink-beam has approximately 10^1^–10^2^ times more photon flux than a monochromatic beam and can provide more reflection information than a monochromatic beam in the same image [2], [3], [4]. Serial crystallography using a pink-beam is advantageous in determining the crystal structure with fewer images when compared with monochromatic beam and being applicable for time-resolved research because of short X-ray exposure [2,3]. The Bragg peaks from the pink-beam serial synchrotron crystallography (SSX) are slightly stretched due to the wide energy bandwidth of the pink-beam. This requires the pink-beam specific indexing algorithm for efficient data processing. Accordingly, the diffraction patterns generated by pink-beam can be useful in developing software for pink-beam data processing. Detailed data collection and processing information are described below.

**Fig. 1:**
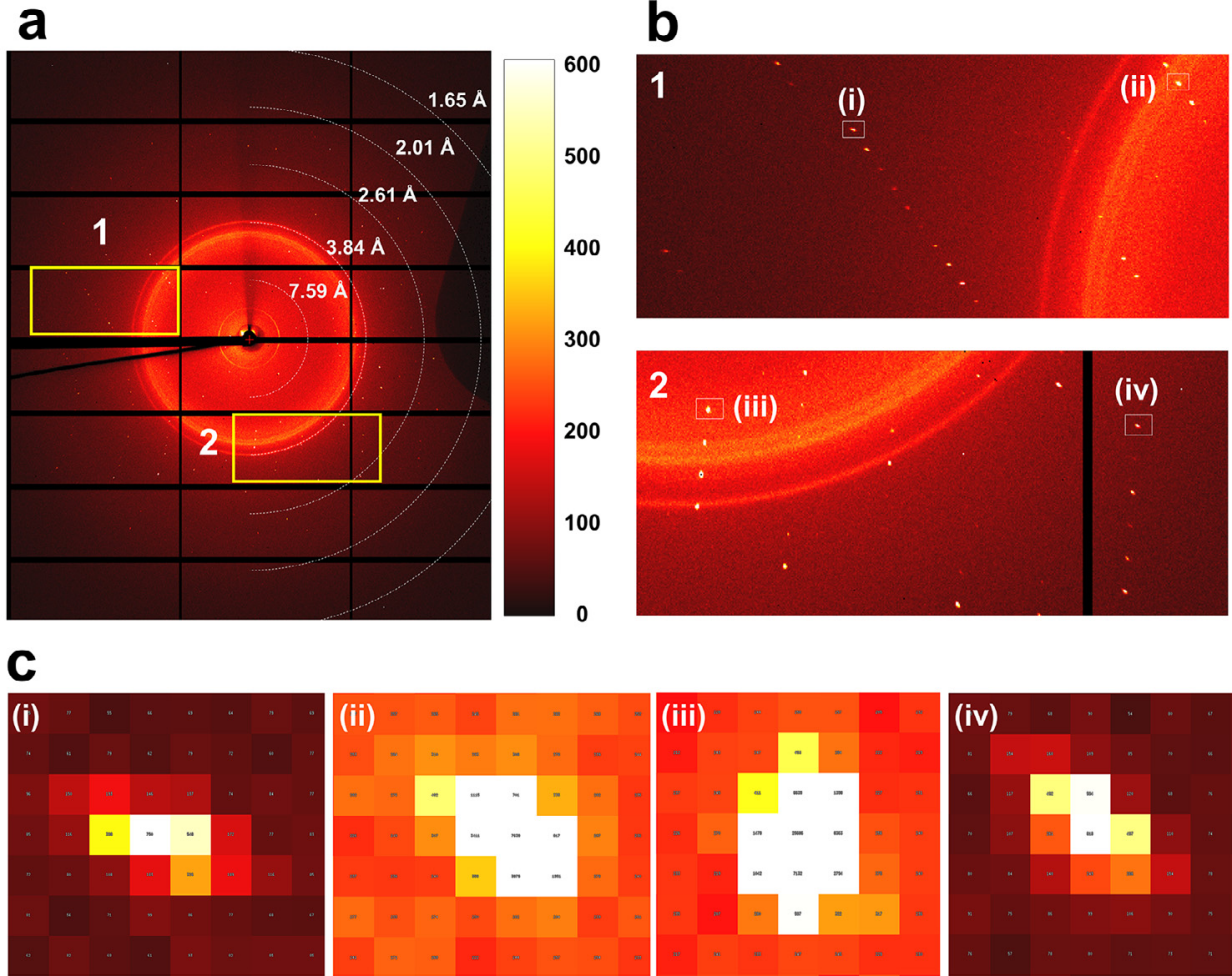
Diffraction images of pink-beam serial synchrotron crystallography (SSX) at 1C beamline at PLS-II. (a) Typical diffraction pattern of lysozyme at pink-beam SSX. (b) close-up view of the diffraction pattern in area of 1 and 2 in Fig. 1a. (c) Close-up view of Bragg peaks in boxes in Fig. 1b.

Pink-beam SSX experiment at beamline 1C at the PLS-II was previously demonstrated [1]. Lysozyme crystals were used as the model sample, which was embedded in a beef tallow injection matrix to deliver the crystals to the X-ray interaction point at a low flow rate. Considering the X-ray beam size (70 × 80 μm, full width at half maximum) used in this experiment, samples were delivered at a high flow rate of 1 μL/min to minimize radiation damage. When the X-rays were exposed to an injection stream comprising a beef tallow injection matrix embedding lysozyme crystals, high X-ray background scattering with 600 analog-to-digital units (ADU) was as observed. This high background scattering occurred because of the large X-ray beam size, the wide thickness of the injection stream (approximately 300 μm), and the inherent properties of the pink-beam. This X-ray background noise potentially reduces the diffraction data quality, such as signal-to-noise ratio. To reduce background scattering and establish the data collection strategy, data was collected following the strategy with different X-ray exposure times and positions on the injection stream as follows: (i) LysEdge50: X-ray exposed to the edge of the injection stream with an X-ray exposure time of 50 ms. (ii) LysCenter50: X-ray exposed to the center of the injection stream with an X-ray exposure time of 50 ms. (iii) LysEdge100: X-ray exposed to the edge of an injection stream with an X-ray exposure time of 100 ms. (iv) LysCenter100: X-ray exposed to the center of the injection stream with an X-ray exposure time of 100 ms. During the pink-beam SSX experiment, all diffraction data sets exhibited Bragg peaks with a slightly stretched shape (Fig. 1).

In theory, samples delivered to the X-ray site will have a cylindrical shape. Accordingly, the center of the delivered sample is approximately 300 μm thick, while the sample thickness reduces toward the edge of the injection stream. Accordingly, background scattering appears differently depending on the X-ray exposure area in the injection stream containing the beef tallow injection matrix; it is lower when X-rays are exposed near the edge rather than the center. Conversely, assuming crystal samples are uniformly present in the same volume as the delivery sample, then more crystal diffraction patterns can be obtained in the center area with a large volume exposed to X-rays. Meanwhile, in the case of X-ray exposure time, when the exposure time is reduced, then photon flux and background scattering can be lowered, as well as the crystal's diffraction intensity. However, the data quality may vary depending on background scattering. The radiation damage suffered by the crystal sample varies with exposure time. Accordingly, two different X-ray exposures were selected to obtain comprehensive information.

Among the data collected through the pink-beam SSX experiment, images with no or weak diffraction intensity were filtered using the Cheetah program. We obtained 22181, 27346, 17532, and 28579 hit images for LysEdge50, LysCenter50, LysEdge100, and LysCenter100, respectively. Pink-beam SSX data were initially processed with different indexing algorithms such as DirAx, XDS, MOSFLM, and XGANDALF. The XGANDALF indexing algorithm used for Bragg peak searching provided the highest indexing rate compared with other applied indexed algorithms. During indexing, detector geometry optimization was performed five times using a *geoptimiser* [5]. Table 1 shows detailed detector geometry information used in data processing.

**Table 1:** Detector geometry information.

Detector geometry parameters	
photon_energy	15,860
clen	280
0/min_fs	0
0/max_fs	1474
0/min_ss	0
0/max_ss	1678
0/corner_x	−687.385
0/corner_y	−834.412
0/fs	+0.999998x +0.001871y
0/ss	−0.001871x +0.999998y
rigid_group_q0	0
rigid_group_a0	0
rigid_group_collection_quadrants	q0
rigid_group_collection_asics	a0
0/coffset	0.001059

**Fig. 2:**
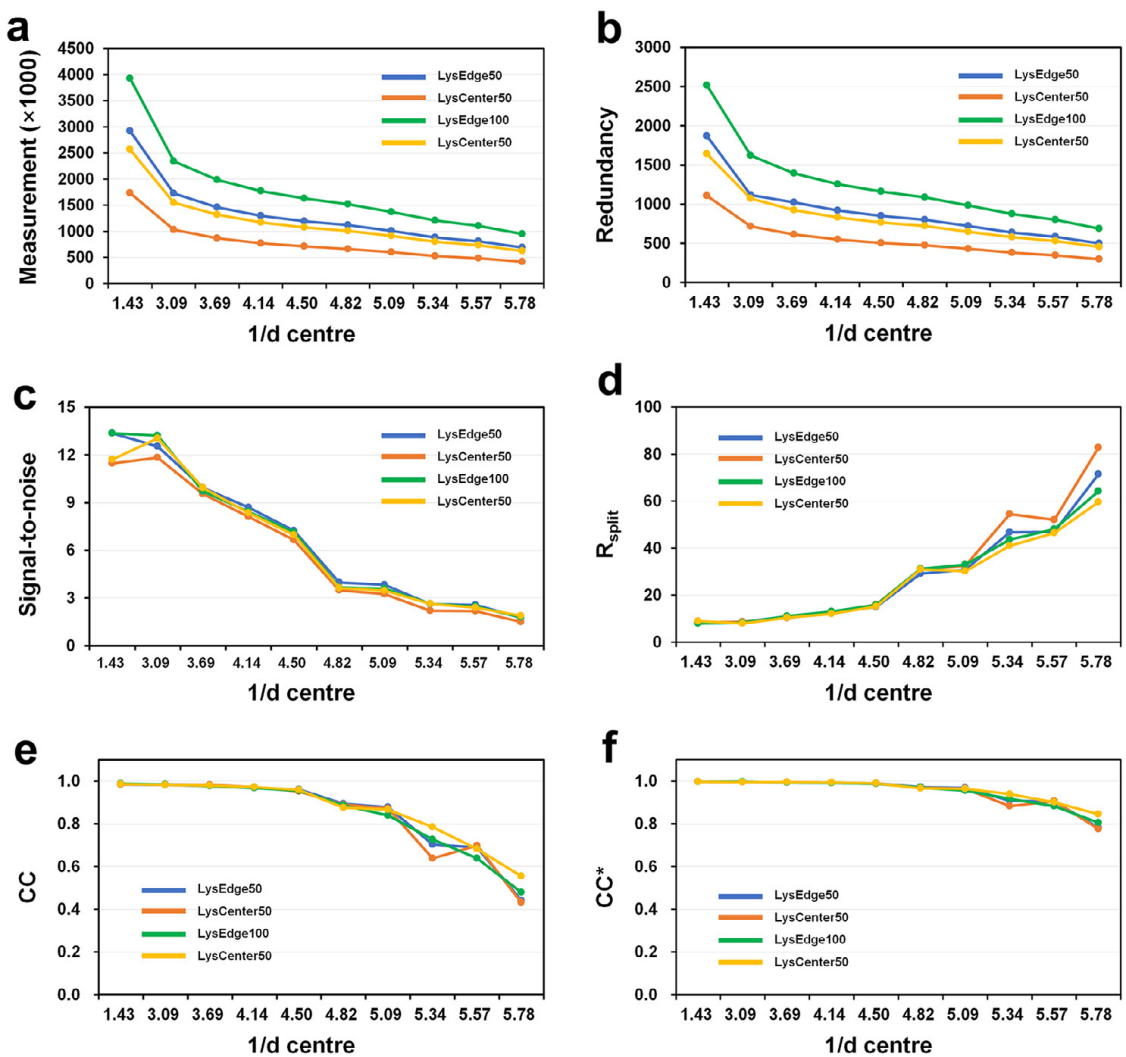
Data processing profiles of LysEdge50, LysCenter50, LysEdge100, and LysCenter100 for (a) measurement, (b) redundancy, (c) signal-to-noise ratio (SNR), (d) R_split_, (e) CC, and (f) CC*.

The indexed images of LysEdge50, LysCenter50, LysEdge100, and LysCenter100 were 17987, 20064, 14410, and 23708, respectively, and the indexing rates were 81.09 %, 73.37 %, 82.19 %, and 82.95 %, respectively. The indexed images included multicrystal hits, and 31109, 31376, 27453, and 43357 indexable diffraction patterns were obtained for LysEdge50, LysCenter50, LysEdge100, and LysCenter100, respectively. The multicrystal hit rates of LysEdge50, LysCenter50, LysEdge100, and LysCenter100 were 72.95 %, 56.37 %, 90.51 %, and 82.87 %, respectively. Table 2 shows the overall SNR and CC of LysEdge50, LysCenter50, LysEdge100, and LysCenter100. Fig. 2 shows the detailed profiles of measurement, redundancy, SNR, Rsplit, CC and CC* of LysEdge50, LysCenter50, LysEdge100, and LysCenter100.

**Table 2:** Data collection and refinement statistics. Processing statistics have been presented elsewhere [1].

Data collection	LysEdge50	LysCenter50	LysEdge100	LysCenter100
X-ray Source	1C, PLS-II	1C, PLS-II	1C, PLS-II	1C, PLS-II
X-ray energy (eV)	15,860	15,860	15,860	15,860
X-ray exposure (ms)	50	50	100	100
Hits images	22,181	27,346	17,532	28,579
Indexed images	17,987	20,064	14,410	23,708
Indexed patterns	31,109	31,376	27,453	43,357
Used patterns	25,000	25,000	25,000	25,000
Space group	P4_3_2_1_2	P4_3_2_1_2	P4_3_2_1_2	P4_3_2_1_2
Cell dimension (Å)				
a	78.88	78.88	78.88	78.88
b	78.88	78.88	78.88	78.88
c	38.05	38.05	38.05	38.05
Resolution (Å)	80.6–1.70 (1.76–1.70)	80.6–1.70 (1.76–1.70)	80.6–1.70 (1.76–1.70)	80.6–1.70 (1.76–1.70)
Unique reflections	14,189 (1380)	14,189 (1380)	14,189 (1380)	14,189 (1380)
Completeness (%)	100.0 (100.0)	100.0 (100.0)	100.0 (100.0)	100.0 (100.0)
Redundancy	925.4 (503.9)	551.7 (300.5)	1256.7 (687.5)	830.0 (455.6)
SNR	6.78 (1.76)	6.13 (1.50)	6.73 (1.79)	6.51 (1.88)
CC	0.9880 (0.4404)	0.9881 (0.4314)	0.9891 (0.4787)	0.9876 (0.5542)
CC*	0.9969 (0.7820)	0.9970 (0.7763)	0.9972 (0.8046)	0.9968 (0.8445)
R_split_ (%)	11.99 (71.60)	12.43 (82.83)	12.23 (64.22)	12.35 (59.72)
**Refinement**				
Resolution (Å)	55.78–1.70	55.78–1.70	55.78–1.70	55.78–1.70
R_work_	21.14 (28.18)	21.77 (30.01)	22.55 (27.90)	22.73 (25.82)
R_free_	23.15 (33.21)	23.41 (37.84)	25.63 (34.56)	25.64 (29.94)
R.m.s. deviations				
Bonds (Å)	0.008	0.015	0.004	0.009
Angles (°)	1.100	1.473	0.758	2.383
*B* factors (Å^2^)				
Protein	22.17	21.02	20.40	19.45
Ligand	26.28	26.33	23.55	22.37
Water	35.43	32.06	32.27	32.23
Ramachandran plot				
Favored (%)	99.21	99.43	99.21	99.21
Allowed (%)	0.79	0.57	0.79	0.79
PDB	8H8T	8H8U	8H8V	8H8W

**Fig. 3:**
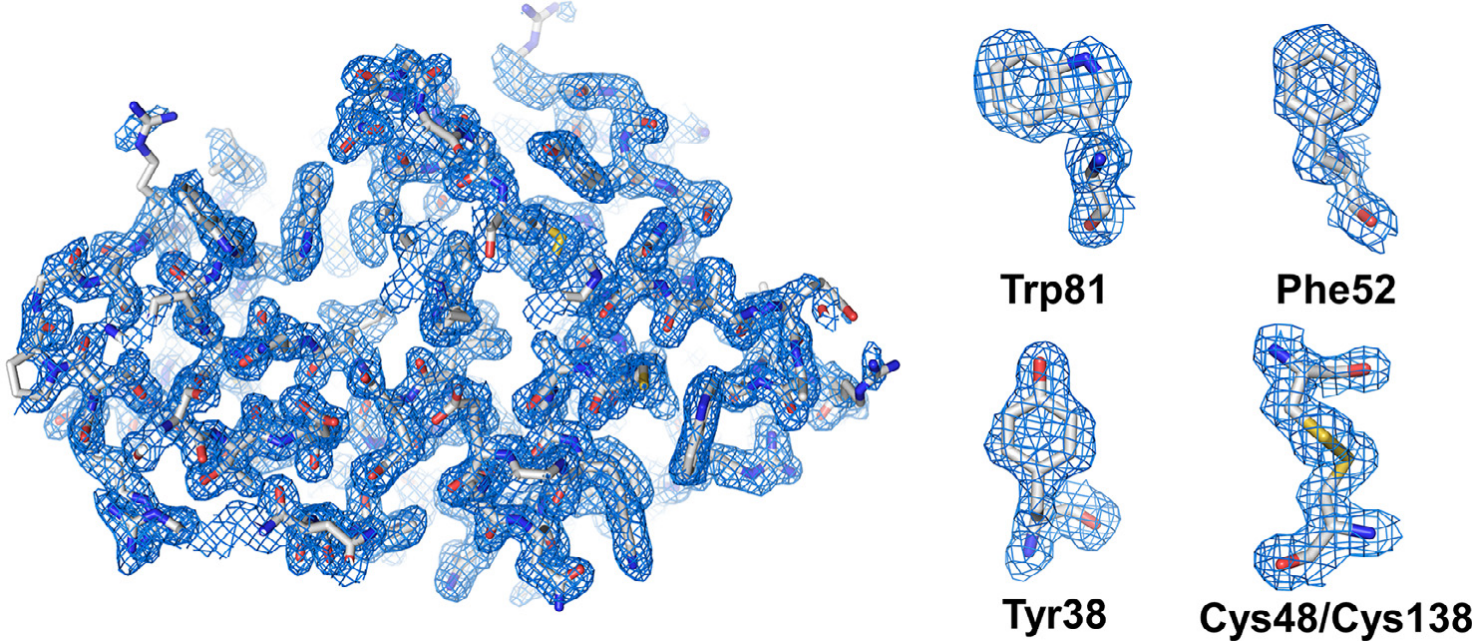
Representative 2fo-fc (blue mesh, 1σ) electron density map of LysEdge50 determined using the pink-beam SSX.

Given that the diffraction patterns obtained from each dataset differ, direct data comparison is impossible. Only 25,000 images were extracted from each dataset, and subsequent data processing was performed. All data were processed up to 1.7 Å. Data exposed to the edge of the X-ray injection stream showed improved SNR in the low-resolution area compared with data exposed to the center. Meanwhile, data with 100-ms X-ray exposure indicated improved CC in high-resolution areas within 2 Å compared with 50-ms exposure. According to the refinement data analysis, the R_work_/R_free_ values of LysEdge50, LysCenter50, LysEdge100, and LysCenter100 were 21.14/23.15, 21.77/23.41, 22.55/25.63, and 22.73/25.64, respectively (Table 2). Refinement statistics indicate that data with less X-ray exposure have better quality than those with more exposure. Electron density maps for all room-temperature structures of lysozyme determined by pink-beam SSX were clearly observed (Fig. 3). According to electron density map analysis, a negative Fo-Fc electron density map, which is considered to be radiation damage at the disulfide bond of lysozyme, was not observed (Fig. 3).

In summary, in this experiment, the pink-beam SSX technique was successfully demonstrated using a lysozyme crystal sample. Furthermore, other protein crystal samples can also be utilized with the pink-beam SSX experiment to determine their room-temperature structures with minimized radiation damage.

## 3 Experimental Design, Materials and Methods

The sample preparation, crystallization, data collection, and processing procedures for pink-beam SSX have been described [1]. Lysozyme crystals were obtained using the batch crystallization method as previously reported [6]. Chicken egg white lysozyme solution (100 mg/mL, 500 L) and crystallization solution (1 mL, 0.1 M sodium acetate (pH 4.4), 5 % (w/v) polyethylene glycol 8000, and 4 M NaCl) were mixed in a microtube. After vortexing at 3000 rpm for 20 s, the mixture was stored at room temperature (24–26 °C). Cubic-shaped lysozyme crystals (20–40 µm) were obtained within one hour, and were delivered to an X-ray interaction point using the syringe pump-based sample delivery method with a viscous medium [7]. Beef tallow injection matrix [8] was used as the sample delivery viscous medium. Furthermore, lysozyme crystal suspension (800 µL) and beef tallow injection matrix (200 μl) were each transferred to a 1-mL BD Luer-Lok™syringe (Sunderland, UK). Both syringes were made in a dual-syringe setup using a coupler. The syringe plunger was moved back and forth more than 30 times to embed the lysozyme crystals in the beef tallow. All mixed samples were transferred to a single syringe. A blunt-tip stainless steel syringe needle (inner diameter: 260 µm) was connected to the syringe containing the sample. The tip of the syringe needle was enclosed with parafilm until data collection to prevent dehydration of the viscous material, including the crystal sample. Pink SSX experiments were performed at beamline 1C at the Pohang Light Source II (PLS-II) in the Republic of Korea. The X-ray energy and bandwidth (ΔE/E) were 15.48 keV and 1.2 %, respectively. The photon flux was approximately 5 × 10^12^ photons/second. The vertical and horizontal X-ray beam sizes (full width at half maximum) were 70 and 80 μm, respectively. A syringe containing the lysozyme crystals embedded in the beef tallow injection matrix was installed into a Fusion Touch 100 syringe pump (Chemyx, Stafford, TX, USA). The crystal samples were delivered to the X-ray interaction point by pushing the plunger via a syringe pump [7], maintaining the flow rate of sample delivery to 1 µL/min. The pink-beam X-ray was continuously exposed to the injection stream without the shutter mode. The diffraction data were collected at ambient pressure and 26 °C ± 0.5 °C. Diffraction data were recorded on a Pilatus3S 2M detector with a 10 Hz (X-ray exposure for 100 ms) or 20 Hz (X-ray exposure for 50 ms) readout. The hit images containing Bragg peaks were filtered using Cheetah [9] with the following parameters: hitfinderAlgorithm= 8, hitfinderMinSNR= 5, hitfinderNPeaks= 30, hitfinderMinPixCount = 2, and hitfinderLocalBgRadius = 2. The diffraction patterns were processed using CrystFEL (version. 0.9.1 + 886ae521) [10] with XGANDALF [11] indexing algorithms. The detector geometry was refined using a *geoptimiser* [5]. Integration and scaling of reflections were performed using a *partialator* in CrystFEL [10]. The electron density map was obtained using molecular replacement methods with Phaser-MR in PHENIX [12]. The crystal structure of lysozyme (PDB code: 7WUC) [13] was used as the search model. Model building and structure refinement were performed using COOT [14] and *phenix.refine* in PHENIX [12], respectively. The final structures were validated using MolProbity [15]. The hit images and geometry files have been deposited in Zenodo (https://zenodo.org/). Structural factors and coordinates have been deposited in the Protein Data Bank (https://www.rcsb.org/).

## Limitations

Not applicable.

## Ethics Statement

This work meets the ethical requirements for publication in this journal. This work does not involve human subjects, animal experiments, or any data collected from social media.

## CRediT authorship contribution statement

**Yongsam Kim:** Data curation, Writing – review & editing. **Ki Hyun Nam:** Data curation, Formal analysis, Validation, Visualization, Writing – original draft, Funding acquisition.

## Data Availability

Diffraction image (50 ms, edge) (Original data) (Zenodo) Diffraction image (50 ms, center) (Original data) (Zenodo) Diffraction image (100 ms, edge) (Original data) (Zenodo) Diffraction image (100 ms, center) (Original data) (Zenodo) Structure factor and coordinates (50 ms, edge) (Original data) (Protein Data Bank) Structure factor and coordinates (50 ms, center) (Original data) (Protein Data Bank) Structure factor and coordinates (100 ms, edge) (Original data) (Protein Data Bank) Structure factor and coordinates (100 ms, center) (Original data) (Protein Data Bank)
